# Papillary Renal Cell Carcinoma in Lynch/Muir-Torre Syndrome with Germline Pathogenic Variant in *MSH6* and Molecular Analysis: Report of a Case and Review of the Literature

**DOI:** 10.15586/jkcvhl.v8i2.175

**Published:** 2021-04-21

**Authors:** Yu Yang, Shweta Dhar, Jennifer Taylor, Bhuvaneswari Krishnan

**Affiliations:** 1 Department of Pathology & Immunology,; 2 Molecular and Human Genetics,; 3 Department of Urology,; 4 Baylor College of Medicine, and Michael E. DeBakey Veterans Affairs Medical Center, Houston, Texas.

**Keywords:** colon adenocarcinoma, Lynch syndrome, MSH6 mutation, Muir-Torre syndrome, Papillary renal cell carcinoma

## Abstract

Lynch syndrome (LS) is an autosomal dominant inherited disorder due to pathogenic variations in the mismatch repair genes, which predisposes to malignancies, most commonly colon and endometrial carcinoma. Muir-Torre syndrome is a subset of LS with cutaneous sebaceous adenoma and keratoacanthoma in addition to the malignancies. Renal cell carcinoma (RCC) in patients with LS is extremely rare. Only 26 cases have been reported and among them, only two cases of papillary RCC. We report a case of synchronous papillary RCC and colonic adenocarcinoma in an 85-year-old male with Lynch/Muir-Torre syndrome. The LS was diagnosed when he presented with multiple sebaceous adenomas and genetic testing showed a pathogenic variant in *MSH6* mismatch repair gene. A colonoscopy at that time showed multiple tubular adenomas with high-grade dysplasia. He was lost to follow-up and presented with gastrointestinal bleeding after 20 years. A right colonic mass, and a solid mass in the lower pole of the right kidney, was detected by imaging. Right Colectomy showed a T3N0 mucin-producing adenocarcinoma. Right nephrectomy showed a T3a papillary RCC which was microsatellite stable with *MSH6*, and *KRAS* mutation. The 36-month follow-up exams showed additional sebaceous neoplasms, and an absence of metastatic carcinoma. Analysis of the reported cases of RCC in LS show clear cell RCC as the most common type. These tumors showed *MLH1* mutation most commonly, unlike the urothelial malignancies in LS which involve *MSH2*. Among the 4 cases of RCC with *MSH6* mutation, three were in females, indicating some gender differences.

## Introduction

Lynch syndrome (LS), an autosomal dominant inherited disorder, is the most common cause of hereditary colorectal carcinoma. It is associated with germline pathogenic variants in the DNA mismatch repair (MMR) genes including *MLH1* (mutL homolog 1), *MSH2/MSH6* (mutS homolog 2 or 6), and *PMS2 (*PMS1 homologue 2). Rarely, it can also be caused by a deletion in the *EPCAM* (epithelial cell adhesion molecule) gene, which leads to epigenetic silencing of *MSH2*. Colorectal and endometrial cancers are the predominant malignancies associated with LS (1, 2). Other malignancies associated with LS include cancers of the stomach, small intestine, breast, ovary, upper urinary tract, bladder, hepatobiliary tract, and brain. The routine screening for genetic colorectal adenocarcinoma by immunohistochemistry (IHC) and/or Microsatellite instability (MSI) testing has led to better detection and an increased understanding of LS. LS accounts for about 3% of newly diagnosed colorectal carcinoma (1) and 3% of newly diagnosed endometrial carcinoma (2). Most commonly, the germline defect involves pathogenic variants in *MLH1* or *MSH2* genes.

A subset of patients with LS develops visceral malignancies associated with cutaneous sebaceous adenomas and keratoacanthoma, which is known as Muir-Torre syndrome (MTS). Although LS and MTS share a defect in one of the 4 MMR genes, a few studies have shown that the frequency of the MMR defect in patients with MTS mostly involves *MSH2* (3). About 5% of the upper urinary tract urothelial carcinomas are associated with LS (4). Recent studies have shown increased frequency of pathogenic variants in *MSH2* in urological malignancies (5, 6).

Cases of renal cell carcinoma (RCC) in LS have been rarely described. An extensive PubMed search revealed 26 cases of possible RCC in LS patients (7–11). Here, we encountered a case of papillary RCC (PRCC) in a LS/MTS patient with germline *MSH6* pathogenic variant. These findings serve to expand the spectrum of urological malignancies in LS and emphasize the need to screen for synchronous and/or metachronous neoplasms in this patient population.

## Clinical History

**Figure 1:**
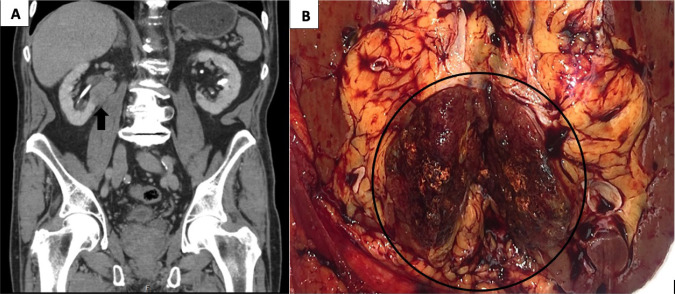
The Computerized Tomography (A) and the gross photograph (B) of the renal mass showing a well circumscribed tan brown mass in the right kidney extending into the renal hilum.

This 85-year-old white male initially presented at the age of 62 years with multiple cutaneous nodules in the head and neck region, diagnosed as multiple sebaceous adenomas. This prompted an investigation for visceral malignancies and genetic testing. Colonoscopy showed multiple tubular adenomas in the descending and sigmoid colon and high-grade dysplasia in the transverse colon. Genetic testing showed a pathogenic variant in *MSH6* Exon 9 (c.3980_3983, dupATCA (p.L1330Vfs’12)). In addition a heterozygous novel variant of uncertain significance (c.8419A>G(p.T2807A) in the *APC* gene was also detected. Since he did not have the typical presentation of polyposis and lack of certainty regarding pathogenicity of the *APC* variant as well as presence of pathogenic variant in the *MSH6* gene, a diagnosis of LS was made. Subsequently, multiple sebaceous adenomas were removed. At the age of 82 years, he presented with rectal bleeding and anemia. Abdominal computerized tomography scan showed a 6.8 × 6.6 × 8 cm mass in the right colon, and a 2.5 × 3.4 × 3.3 cm solid mass on the lower pole of the right kidney involving the hilum (Figure 1A), multiple hypodense nonenhancing lesions involving both lobes of the liver, and a 2.5 × 2.8 cm hypodense lesion in the body of the pancreas. The subsequent subtotal colectomy showed a T3N0 mucin-producing adenocarcinoma in the cecum. The wedge biopsy of the liver lesion at the same time showed a sclerosed hemangioma. The renal mass biopsy showed a PRCC. Three months later, a right nephrectomy was performed. Clinical follow-up 3 years after the resection shows multiple additional sebaceous adenomas involving the head and neck area and sebaceous carcinoma involving the eyelid. The pancreatic lesion is stable, and a biopsy was not performed. The timeline of the various tumors in this patient is shown in Table 1. There is no evidence of metastatic colon or RCC.

**Table 1:** List of neoplasms in this patient.

Year	Tumor site	Tumor type
1998	Scalp, temple, nose Transverse colon	Sebaceous adenoma, skin squamous carcinoma Tubular adenoma with high-grade dysplasia and multiple tubular adenoma
2007	Chest	Sebaceous adenoma
2010	Nose	Sebaceous adenoma and squamous carcinoma
2014	Neck	Sebaceous adenoma
2015	Cheek	Sebaceous adenoma
2016	Scalp	Sebaceous adenoma
2017	Cecum and right colon Kidney	Adenocarcinoma of cecum and tubulovillous adenoma with high-grade dysplasia of right colon Papillary renal cell carcinoma
2018	Scalp, cheek, philtrum	Sebaceous adenoma
2019	Shoulder Lower tarsal conjunctiva	Sebaceous adenoma Sebaceous carcinoma
2020	Right shoulder	Sebaceous adenoma

## Pathologic Evaluation

**Figure 2:**
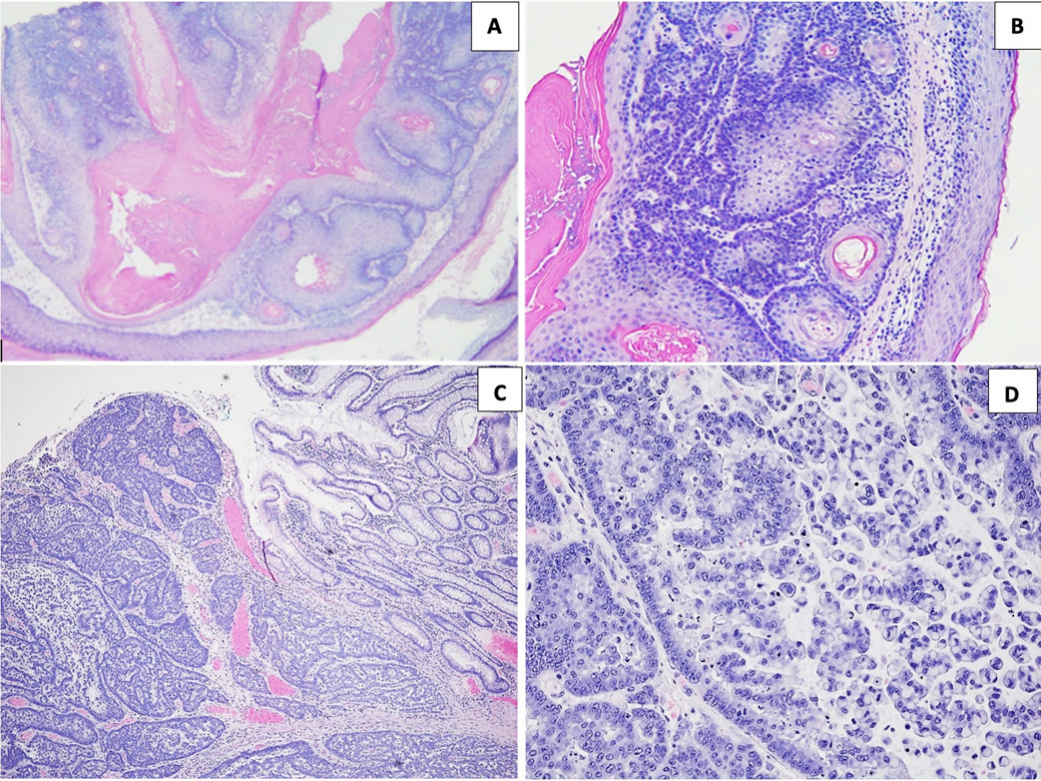
(A) Sebaceous adenoma in the dermis with cystic dilatation (Hematoxylin and Eosin ×40). (B) Proliferation of germinative cells in the sebaceous adenoma (Hematoxylin and Eosin ×200). (C) Low magnification view of the colonic adenocarcinoma with normal colon on the right and the adenocarcinoma with mostly solid architecture (Hematoxylin and Eosin ×40). (D) Poorly differentiated adenocarcinoma of colon with pleomorphic tumor cells and poorly formed glands with some extracellular and intracellular mucin (Hematoxylin and Eosin ×200).

The multiple sebaceous adenomas histologically showed dermal tumors with varying proportions of basaloid cells and sebaceous cells with cystic changes (Figure 2A and B). The pathologic evaluation of the colonic tumor showed a 7.5 × 7.2 × 4.3 cm fungating mass within the cecum. Histologically, the tumor was a high-grade adenocarcinoma with mucin production and invading the subserosal adipose tissue. Tumor infiltrating lymphocytes or germinal centers were not seen (Figure 2C and D). Strong nuclear expression of MLH1, PMS2, and MLH2 mismatch repair proteins was seen in the adenocarcinoma by IHC. Faint MSH6 was expressed in about 3% of the nuclei. The tumor was staged as T3N0.

**Figure 3:**
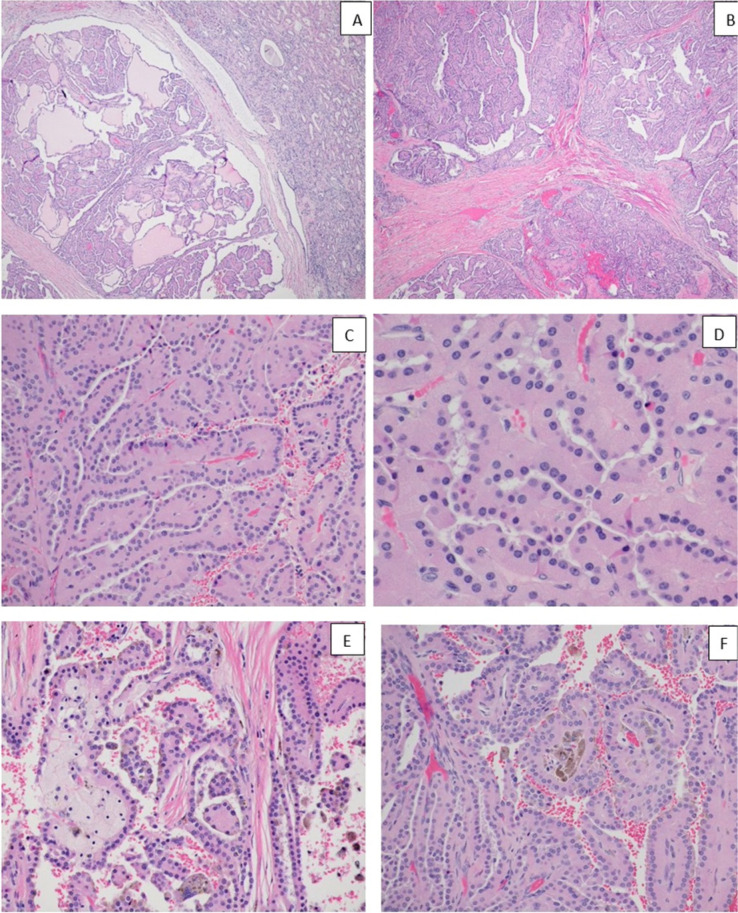
Hematoxylin- and eosin-stained sections of the renal tumor (A). Low magnification showing a well-circumscribed tumor with papillary structures and normal kidney on the right (×40) (B). Well-circumscribed papillary tumor with fibrous septa separating the tumor (×40) (C). Papillary architecture of the tumor with thin fibrovascular core (×100) (D). The tumor cells have abundant granular eosinophilic cytoplasm and a round nucleus placed away from the basal lamina (×400) (E). Papillary tumor with foamy histiocytes (×200) and F. Papillary tumor with hemosiderin pigment (×200) (F).

**Figure 4:**
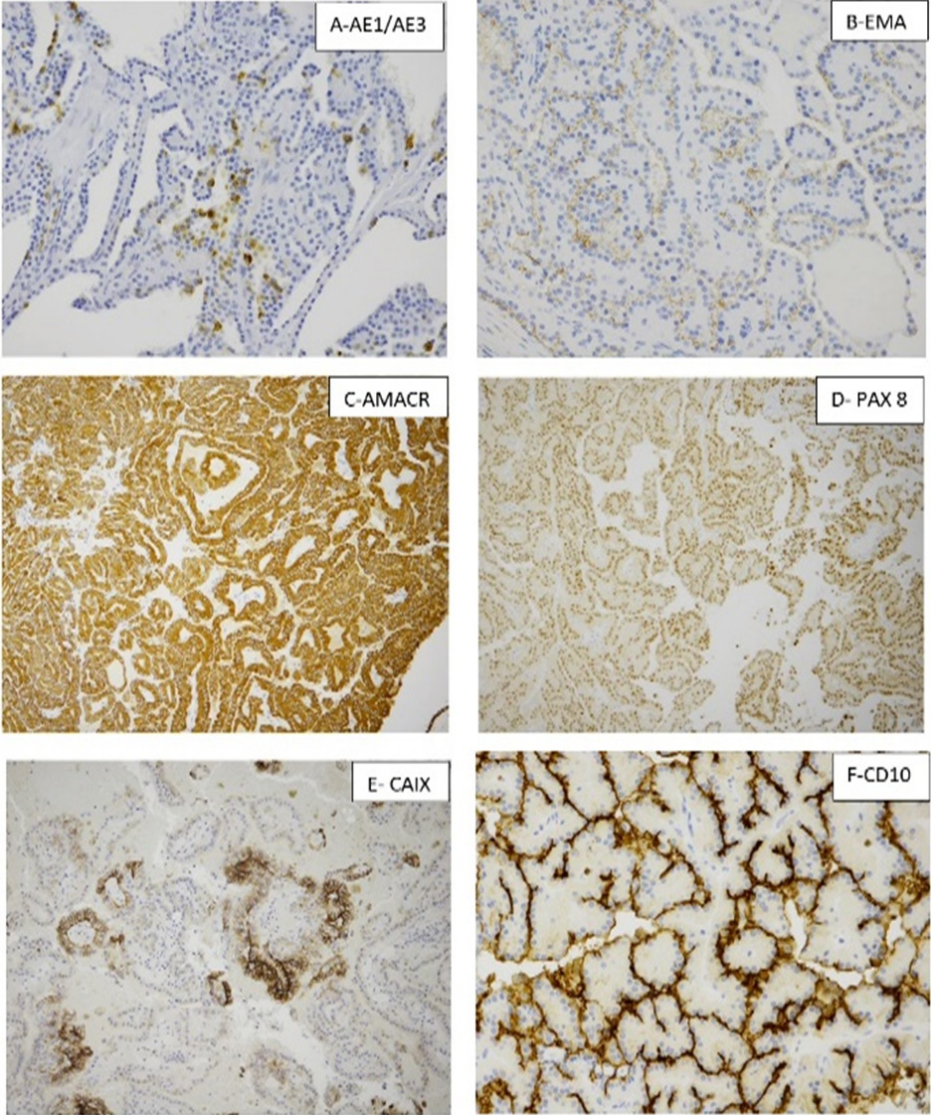
Immunohistochemical stains showing (A) Focal staining for AE1/AE3. (B) Membranous staining for epithelial membrane antigen (EMA). (C) Diffuse cytoplasmic staining for AMACR. (D) Diffuse nuclear staining for PAX8. (E) Focal staining for CAIX. (F) Diffuse strong luminal membranous staining for CD10.

**Figure 5:**
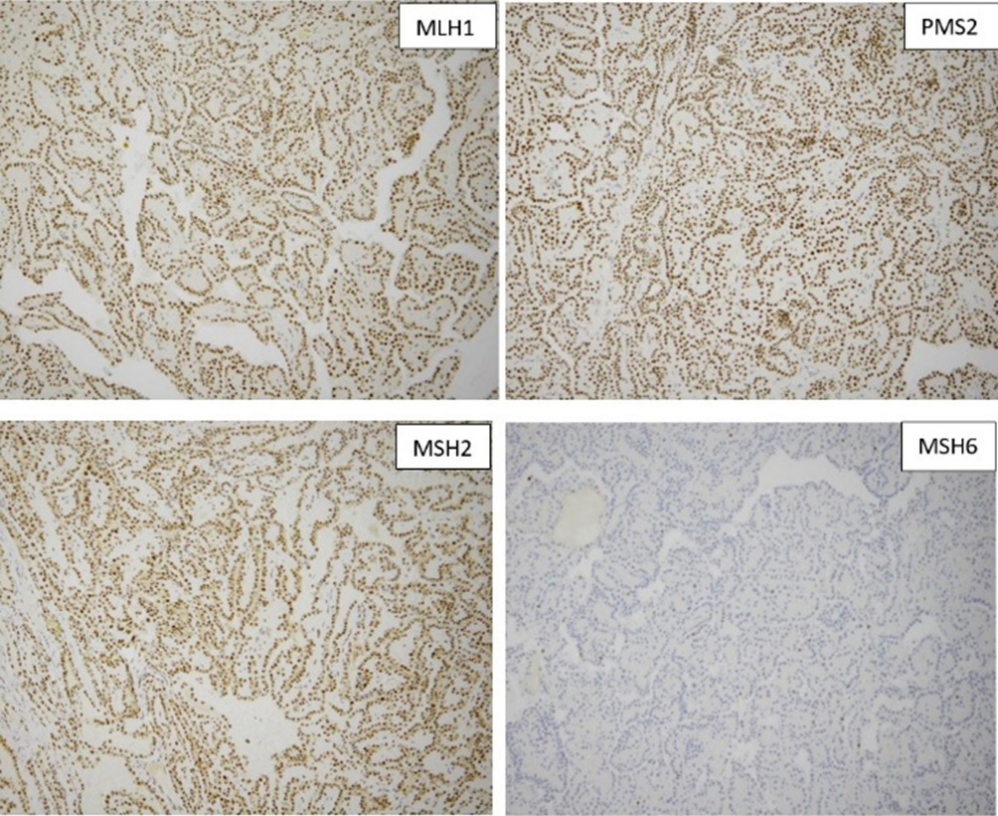
Immunohistochemical stain for mismatch repair proteins showing loss of expression of MSH6 and preserved nuclear expression of MLH1, PMS2, and MSH2.

The pathological evaluation of the kidney showed a 3.4 × 3.3 × 3.0 cm well-circumscribed solid brown mass in the lower pole, extending into the hilum and perinephric fat (Figure 1B). Histologically, the tumor showed a mass without a capsule blending with the uninvolved renal parenchyma. Dense sclerotic stroma was present between clusters of tumor cells. The neoplasm showed a 90% papillary architecture with thin fibrovascular core, and occasional foamy histiocytes. The papillae were lined by a single layer of columnar cells with an abundant eosinophilic granular cytoplasm. The nucleus was low grade, round, and uniform with fine chromatin, placed away from the basal lamina without pseudostratification. The nuclear features were classified as ISUP grade 2. Focal areas of hemorrhage and cytoplasmic hemosiderin pigment in the tumor cells were present. Psammomatous calcification was absent (Figure 3A–F). IHC showed focal patchy positivity for AE1/AE3 and diffuse membranous staining for epithelial membrane antigen (EMA). There was a strong, diffuse cytoplasmic expression of vimentin, AMACR, luminal membranous expression of CD10, and nuclear expression of PAX8 (Figure 4A–F). The CAIX stain showed focal positivity. Cytokeratin 7 (CK7), high molecular weight cytokeratin, GATA3, and CD117 stains were negative in the tumor cells. These immunohistochemical features were most consistent with PRCC, type 2. IHC for mismatch repair proteins showed faint expression of MSH6 (1% of nuclei) and strong nuclear expression of MLH1, MSH2, and PMS2 (Figure 5A–D). The tumor was staged as T3a with sinus and perinephric fat invasion. Vascular invasion was not seen.

The Foundation Medicine next-generation sequencing (NGS) analysis of the renal tumor was performed. The genomic abnormalities included *ARID1A* S90fs*11, *MSH6* L1330fs*12, *SETD2* N1725fs*3, and *STAG2* E143fs*3. In addition, variants of unknown significance (VUS) were detected in the tumor. These included *APC, EGFR, FANCC, FGF14, KDM6A, KRAS, MLL2*, and *REL*. The renal tumor was microsatellite stable.

## Discussion

There has been an increasing understanding of LS attributed to pathogenic variants in one of the four mismatch repair (MMR) genes, namely, *MLH1, MSH2, MSH6*, and *PMS2*. Pathogenic variants in the MMR genes lead to abnormal microsatellite sequences referred to as MSI. The National Cancer Institute recommends five markers as the screening tool for MSI. These include three dinucleotide repeats (D2S123, D5S346, and D17S250) and two mononucleotide repeats (BAT25, BAT26). Identification of three of the five markers is considered positive for MSI (3). The MSI predisposes to the development of multiple malignancies. The malignancies associated with LS commonly include colorectal carcinoma and endometrial cancer. Other malignant sites include breast, ovary, stomach, small bowel, urinary tract, hepatobiliary tract, and brain. A subset of LS patients also presents with cutaneous sebaceous adenomas and keratoacanthoma, called the MTS. Analysis of LS patients with cancer has shown that the germline variants involve *MLH1* and *MSH2* genes in 60–80% of the cases. Only a minority of cases involves *MSH6* and *PMS2* (12). On the other hand, germline variant analysis in patients with MTS showed the involvement of *MSH2* in 89% of the cases and *MLH1* in 11% of the cases (3). Similarly, germline variant analysis in urothelial carcinoma patients with LS has shown frequent involvement of *MSH2* and *MSH6* (5, 6). These studies indicate that the clinical spectrum of malignancies depends upon which gene is involved in the pathogenesis. The increasing understanding of the clinical manifestations can lead to appropriate screening and prevention strategies.

RCC is uncommonly reported in LS. Extensive literature search revealed 26 other cases of possible RCC in LS patients (7–11). The details of these cases are shown in Table 2. There were nine clear cell RCC and three cases of PRCC including the current case. Histological subclassification is not available in 15 cases. Clear cell RCC appears to be the most common tumor type in LS. The age at presentation ranged from 47 to 82 years among men and 57–67 years among women. The incidence of these tumors in female patients is much higher than the sporadic tumors with a male to female ratio of 1:1 compared to 2:1 in the general population. Unlike the upper tract urothelial carcinoma, which are more commonly associated with *MSH2* variants, RCC was associated with *MLH1* variants, which was seen in 16 cases (57.1%). Variants in *MSH2* were seen in 7 cases (25%) and in *MSH6* in 4 cases (14.8%). Interestingly, three out of four patients with the *MSH6* variants were females. In the study by Therkildsen et al., the incidence of RCC was higher in the Danish patients with LS compared to the general population (10). This was not seen in the study from Finland (7).

**Table 2:** Reported cases of renal cell carcinoma in Lynch syndrome.

Case	Age/Sex	Mutation type	Renal cell carcinoma	Stage/grade	Other tumors	Ref
1	77/NP	MLH1, ex 16	RCC, subtype NP	T1,N0,G2	NP	(7)
2	55/NP	MLH1, ex 16	RCC, subtype NP	T2,N0,G2	NP	(7)
3	56/NP	MLH1, ex 16	RCC, subtype NP	T3,N0,G2	NP	(7)
4	88/NP	MLH1, ex 16	Not resected		NP	(7)
5	62/NP	MLH1, ex 16	RCC, subtype NP	T1,N0,G2	NP	(7)
6	62/NP	MLH1, ex 16	RCC, subtype NP	T3,N1,G2	NP	(7)
7	65/NP	MLH1, ex 3	RCC, subtype NP	T1,N0,G2	NP	(7)
8	64/NP	MLH1, ex 6	RCC, subtype NP	T1,N0,G1	NP	(7)
9	60/NP	MLH1, ex 16	RCC, subtype NP	T1,N0,G3	NP	(7)
10	47/NP	MLH1, ex 6	RCC, subtype NP	T3,N0,G2	NP	(7)
11	65/F	MSH6 (NP)	Clear cell (No MTS)	NP	NP	(8)
12	60/F	MSH6(3311delTT)	Clear cell	T1b,Nx,G2	Colon and endometrium	(9)
13	67/F	MSH2 c.1750+ST>A	Clear cell	T1b	Colon	(10)
14	61/M	MSH2 c.368delC	Clear cell	T2	Colon	(10)
15	50/M	MSH2 c.368delC	Clear cell	T2	None	(10)
16	61/M	MSH2 c.1176C>T	Papillary	T1a	Colon, ureter, and renal pelvis	(10)
17	66/F	MLH1 c.1852_1854delAAG	Clear cell	T3a	None	(10)
18	47/M	MLH1 c.1667+2delTCAinsATTT	Clear cell	T1a	None	(10)
19	61/M	MLH1 c.1667+2delTCAinsATTT	Clear cell	T2	Gastric	(10)
20	62/F	MSH6 c.1444C>T	Clear cell	T3a	Ovarian	(10)
21	82/M	MLH1 c.1732-2A>T	NP	NP	Skin, colon, and small bowel	(10)
22	58/M	MLH1 c.1852_1854delAAG	NP	NP	Colon	(10)
23	59/F	MLH1 c.1667+2delTCAinsATTT	NP	T3a	Endometrium	(10)
24	82/M	MSH2 c.942+3A>T	NP	NP	Colon	(10)
25	57/F	MSH2 c.892C>T	NP	NP	None	(10)
26	61/F	MSH2, but no loss of expression of MMR protein in papillary RCC	Papillary, type 2	T1a	Ureter, colon, and endocervix	(11)
27	85/M	MSH6 Exon 9 (C3980-3983, dup ATCA)	Papillary	T3a	Colon, sebaceous adenoma, and sebaceous carcinoma	This case

NP: Not provided

The *MSH6* gene is located in the short arm of chromosome 2, close to *MSH2*. Abnormalities in the *MSH6* gene were first discovered in studies involving hereditary nonpolyposis colorectal cancers, from 5 Japanese families who did not fulfill the Amsterdam criteria and had no pathogenic variants in *MLH1* and *MSH2* but had a family history of endometrial and ovarian cancers. Population-based studies indicate higher pathogenic allele frequency rate for *MSH6* and *PMS2*, but with variable penetrance (13). *MSH6* is not involved in the repair of dinucleotide repeats and only the mononucleotide repeats are involved. When a pathogenic variant in the *MSH6* gene is suspected, a panel of five mononucleotides (NR21, BAT25, BAT26, NR24, and NR22) is recommended to assess the MSI status (3). There have been relatively few publications of isolated *MSH6* variants. A large study of 113 families with *MSH6* variant carriers from multiple countries has shown some differences in presentations and in malignancies associated with *MSH6*. The presentation of malignancies is at a much later age extending to 70 and 80 years as seen in our patient who presented with the adenocarcinoma at the age of 82 years. These studies suggest long-term screening requirement for patients with the *MSH6* pathogenic variant (13, 14). Endometrial carcinoma is the most common presentation among women with pathogenic variants in this gene, and the women have a sixfold increased incidence of other cancers associated with LS including ovary, stomach, kidney, ureter, or brain (14). By the age of 70 years, 24% of men and 40% of women will be diagnosed with LS-associated malignancy and the incidence increases to 47 and 65% by the age of 80 years in men and women, respectively. The reported studies also show occurrence of other malignancies such as lymphoma, leukemia, Langerhan’s cell histiocytosis, and testicular germ cell tumors with *MSH6* variants (3, 15). These studies suggest a different set of malignancies must be considered in the screening of patients with *MSH6* pathogenic variant.

There were six cases of kidney tumors in the study of pure *MSH6* variants by Baglietto et al. (14), but the type of tumor, whether urothelial carcinoma or RCC, was not specified. The age of presentation of renal tumors ranged from 39 to 75 years, indicating earlier presentation than the sporadic tumors in some patients. It is also interesting to note that four of the six renal tumors in this report were among women. Similarly, in our review, there were four RCCs associated with *MSH6* variants including the current case. There were three cases of clear cell RCC, all in females, none of whom had MTS (8–10). This current report is the only case of PRCC in a male with MTS and a pathogenic variant in *MSH6*. This indicates that PRCC in these patients is extremely uncommon. It is also possible that other sporadic tumors may arise in these patients that are not generally seen in LS. However, the loss of expression of the MSH6 protein by IHC and the *MSH6* variant in this papillary tumor as confirmed by the tumor NGS analysis, confirms this to be definitively associated with *MSH6*.

There have been only three reports of PRCC in LS including the current case. The two reported cases of PRCC were associated with urothelial carcinoma of the ureter and/or renal pelvis. In one case with LS due to the *MSH2* variant, loss of the MSH2 expression and MSI were present in the colon, ureter, and cervical adenocarcinoma, but the PRCC of the kidney was microsatellite stable and did not show any loss of the MMR proteins. Histologically this tumor was described as type 2 PRCC that was confined to the kidney, but a photograph of the tumor or the background uninvolved kidney was not available (11). In the second case of papillary carcinoma, the histomorphology of the tumor was not described or photographed. It is possible that this papillary carcinoma was arising in the background of an atrophic/inflamed kidney since both these cases had associated urothelial carcinoma of the ureter/renal pelvis (10, 11). The PRCC in the current case was in a normal functioning kidney without an urothelial carcinoma. Our case is the first report of PRCC associated with pathogenic variants in *MSH6* in LS/MTS. The *MSH6* abnormality (L1330fs*12) was also seen in the papillary carcinoma. Other abnormalities included *ARID1A* S90fs*11, *SETD2* N1725fs*3, and *STAG2* E143fs*3. In addition, VUS were detected in the tumor in genes such as *APC, EGFR, FANCC, FGF14, KDM6A, KRAS, MLL2*, and *REL*.

Unlike the RCC with clear cytoplasm, the papillary renal tumors have been difficult to classify based on light microscopic and immunohistochemical features. In the 2016 WHO classification of renal tumors, the PRCC was subclassified into types 1 and 2 as previously reported by Delahunt et al. The type 1 PRCC is a well-defined entity showing papillary tumor lined by low cuboidal cells with basophilic or amphophilic cytoplasm. The type 2 PRCC shows pseudostratified epithelial cells with abundant eosinophilic cytoplasm and high-grade nuclei with prominent nucleoli. In the WHO classification, it was also acknowledged that the type 2 PRCC is a heterogeneous group of tumors. The type 2 PRCC must be differentiated from other RCCs with papillary architecture. These include hereditary leiomyomatosis and RCC (HLRCC), collecting duct carcinoma, renal medullary carcinoma, and MiT family translocation carcinoma. HLRCC typically shows a PRCC with prominent nucleolus and a perinucleolar halo. These tumors show loss of Fumarate hydratase and overexpression of 2SC by IHC. The collecting duct carcinoma shows an infiltrating tumor in a desmoplastic stroma. The expression of OCT3/4, SMARCB1, and p63 differentiates it from type 2 PRCC. The renal medullary carcinoma is associated with a clinical history of sickle cell trait and shows loss of SMARCB1 by IHC. The MiT family translocation carcinomas include tumors with gene fusions involving the TFE3 or TFEB locus. The diagnosis is made by TFE3 or TFEB break apart fluorescent in situ hybridization (FISH) assay, which is more sensitive than the immunohistochemical stains for TFE3 and TFEB.

The oncocytic PRCC was described by many authors (16–19), and it was not included in the WHO classification, needing further characterization. It has been acknowledged by many authors (20, 21) that often PRCC shows features of both types 1 and 2 with significant interobserver variability making the subclassification more difficult. The comprehensive study by Saleeb et al. (21) subtyped the PRCC into four types PRCC1–PRCC4 based on histologic, immunohistochemical, and molecular findings. The PRCC1 and PRCC2 corresponded with the WHO type 1 and 2, respectively. The PRCC3 had features of both types 1 and 2, while PRCC4 had features of oncocytic PRCC. This same study also demonstrated that the PRCC4 or oncocytic PRCC is positive for GATA3 by IHC. Al-Obeidy et al. (22), while reviewing the oncocytic PRCC, discovered a distinct subtype of papillary renal tumors with papillary or tubular architecture lined by a single layer of cuboidal cells with eosinophilic granular cytoplasm, with apically located round nucleus away from the basal lamina without pseudostratification, which was called a papillary renal neoplasm with reverse polarity. These tumors showed a male to female ratio of 1:1, ISUP grade 1–2 tumors without metastasis after a follow-up of 20 months. Unlike types 1 and 2 PRCC, these tumors were positive for GATA3 and negative for vimentin. The next-generation sequencing of 10 of these tumors performed by the same group showed KRAS missense variants in eight tumors. This variation was absent in 30 types 1 and 2 PRCC that were studied (23). One case of GATA3-positive papillary renal neoplasm with reverse polarity with KRAS and PIK3CA mutation is also described by Lee et al. (24). Similarly, KRAS mutations were also observed by Kim and Tong et al. (25, 26) who have described KRAS mutations in papillary renal neoplasm with reverse polarity, but GATA3 immunohistochemical stain was not performed in these two studies. In the study by Bayrak et al. (27) KRAS mutation was not detected in the CCRCC, Chromophobe RCC, and PRCC. The subtype of PRCC examined was not described. These studies indicate that the KRAS mutation is seen in oncocytic papillary neoplasms.

The PRCC in this study did not particularly fit into a specific type of PRCC. It showed some light microscopic features of papillary renal neoplasm with reverse polarity, including a papillary architecture with oncocytic cells showing apically placed low-grade nuclei without pseudostratification. Interestingly, a KRAS variant was detected in this tumor as in papillary tumors with reverse polarity. Follow-up evaluations 36 months after the nephrectomy show the absence of metastatic disease. This tumor differed from papillary renal neoplasm with reverse polarity by the presence of rare collections of histiocytes in the papillary fibrovascular cores. Immunohistochemically, strong expression of vimentin and absence of GATA3 and only focal staining for cytokeratin AE1/AE3 was also not compatible with papillary renal tumor with reverse polarity. The immunohistochemical features were that of type 2 PRCC. The presence of KRAS mutation in this tumor suggests that this mutation may not be restricted to the papillary renal neoplasm with reverse polarity and this entity should be better defined with the study of large number of cases.

It is well-known that LS patients may have multiple synchronous or metachronous malignancies (28). In our case, mucin-producing colonic adenocarcinoma was synchronous with PRCC. Interestingly, it has been reported that patients with both colorectal and RCC diagnoses have an increased risk for additional malignancies, regardless of whether the patient has a hereditary cancer syndrome or not (29). Because of the rarity of this entity, the pathologic behavior is yet to be determined. But, it emphasizes the importance of careful follow-up in such a patient population.

Few unusual and uncommon tumors have also been reported in LS, including adrenocortical carcinoma, peritoneal mesothelioma (30, 31), pancreatic acinar carcinoma, pancreatic neuroendocrine carcinoma (30), low-grade serous carcinoma, leiomyosarcoma, liposarcoma, malignant fibrous histiocytoma, anaplastic thyroid carcinoma, melanoma, breast carcinoma, and prostatic adenocarcinoma (31, 32). Whether all these tumors like the renal tumors in this study are truly related to LS or are coincidental tumors in a background of MSI needs to be determined. The study of the molecular profiles of different LS-associated tumors by Gylling et al. (33) showed that LS-associated tumors developed by various routes. The level of MSI was variable in the different LS-associated tumors, with high MSI in ureter, stomach, and colonic carcinomas and much lower in renal and brain tumors. Similarly, the papillary tumor in this case was microsatellite stable. Further studies are necessary for a better understanding of the development of these uncommon tumors.

## Conclusion

We describe the first case of PRCC in a patient with Lynch/MTS with the *MSH6* pathogenic variant. The PRCC showed oncocytic cytoplasm with reverse nuclear polarity, and loss of MSH6 by IHC and NGS. There was also KRAS mutation, but the tumor was microsatellite stable. This tumor had some features of the recently described papillary renal neoplasm with reverse polarity, including the KRAS mutation, but it was immunohistochemically like type 2 PRCC, suggesting that the KRAS mutation is not unique to papillary renal neoplasm with reverse polarity. Including the current case, 27 cases of RCC have been reported in association with LS.
